# Multiple Focal Brown Tumors (Osteitis Fibrosa Cystica) in a Renal Transplant Recipient

**DOI:** 10.1155/2022/4675041

**Published:** 2022-03-07

**Authors:** Thavathurai Priyanthan, Anne Pernille Hermann, Jonas Asgaard Bojsen, Anne Bruun Krøigaard, Claus Bistrup, Erik Bo Pedersen

**Affiliations:** ^1^Department of Nephrology, Odense University Hospital, Odense, Denmark; ^2^Department of Endocrinology, Odense University Hospital, Odense, Denmark; ^3^Department of Clinical Research, University of Southern Denmark, Odense, Denmark; ^4^Department of Radiology, Odense University Hospital, Odense, Denmark; ^5^Department of Pathology, Odense University Hospital, Odense, Denmark

## Abstract

Brown tumors (BTs) are manifestations of osteitis fibrosa cystica that develops due to increased osteoclast activity secondary to hyperparathyroidism (HPTH). The name comes from its characteristic brown color due to high hemosiderin level and hemorrhage surrounded by osteoclastic giant cells, fibrous tissue, and bone fragments. Presentation can be either unifocal or rarely multifocal. Misdiagnosis of BT compared to malignant giant cell tumor is not uncommon. Early diagnosis and intervention may prevent destructive bone changes. Treatment of BTs due to chronic renal failure should be aimed primarily at its prevention with phosphate binders, vitamin D (analogues), calcimimetics, and prolonged dialysis sessions. Parathyroidectomy can be the option in nonresponsive cases. In this report, we present an unusual case of multiple brown tumors in a 54-year-old female renal transplant patient involving the spine, jaw, and scapula, initially misdiagnosed as giant cell tumor. Five years later, the patient was diagnosed with BT because of the medical history, morphology, and negative p63 staining in combination with secondary/tertiary hyperparathyroidism. The patient subsequently underwent subtotal parathyroidectomy.

## 1. Introduction

Brown tumors (BTs) were first described by Friedrich Daniel von Recklinghausen in 1891 [1]. They are seen in osteitis fibrosa cystica (OFC), where giant cell focal lesions consisting of osteoclasts and fibrous tissue give rise to lytic skeletal lesions. The name comes from its characteristic brown color due to the high hemosiderin level from the collection of osteoclastic giant cells, fibrous tissue, and blood in the surrounding stroma. They are well documented in both primary and secondary/tertiary hyperparathyroidism (HPTH) and occur due to increased osteoclast activity. Prevalence of these tumors is lower in secondary HPTH (1.5–1.7%), compared to primary HPTH (3%) [2–4]. Presentation can be either unifocal or rarely multifocal [3]. BTs have been reported in various locations such as jaw bones, ribs, and vertebrae [2, 4, 5]. BTs have no malignant potential; however, they can present as expansive lesions compressing surrounding structures or produce facial disfigurement [3, 4, 6].

The incidence of these tumors in dialysis patients is unknown but increasing due to longevity of these patients [2, 3, 7]. Solitary BTs have been reported previously in renal transplant patients [8]; in this report, we present an unusual case of multiple focal brown tumors in a renal allograft recipient at predialysis stadium involving the spine, initially misdiagnosed as giant cell tumor.

## 2. Case Report

**Figure 1:**
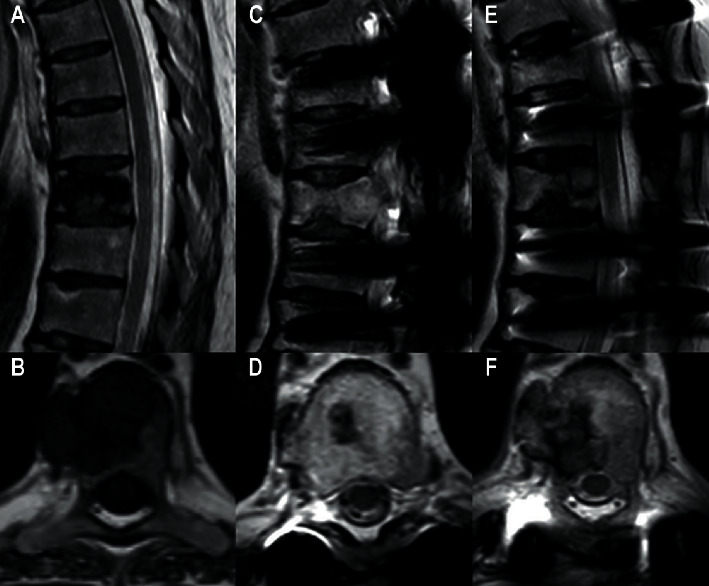
Spine MRI. (a, b) Sagittal T2 weighted Dixon in-phase and axial T1 weighted sequences from initial spine MRI showing tumor in the body of Th8. (c, d) Sagittal T2 weighted Dixon in-phase and axial T2 weighted sequences performed 19 months later showing spondylodesis and partial regression of tumor. (e, f) Sagittal and axial T2 weighted sequences performed additionally 33 months later showing progression of tumor.

A 54-year-old female with a 35-year history of type-1 diabetes mellitus, complicated by retinopathy (blindness), neuropathy (Charcot's foot, gastroparesis), and chronic kidney failure, received a deceased-donor renal allograft in 2002. She was on triple immunosuppressive therapy including cyclosporine A, mycophenolate mofetil, and prednisolone. Thirteen years posttransplant, she presented with abdominal pain; a positron emission tomography-computed tomography (PET-CT) revealed several fluorodeoxyglucose (FDG) absorbing bone lesions corresponding to the transverse process of the thoracic vertebra (T12), the body of T8, and the scapulae bilaterally. Subsequent magnetic resonance imaging (MRI) confirmed the tumors in T8 and T12, each measuring approximately 3 cm with a T2-weighted hypointense lesion with paravertebral expansion (Figure 1). Reevaluation of previous scans brought attention to changes in the jaw bones. Specifically, no pathological lymph nodes were seen. A biopsy from T12 suggested a giant cell tumor, although a final diagnosis was not established.

No progression was seen during the insuring three years on serial MRI of the spine. However, 44 months following the initial MRI, the patient developed irregular bone changes in her left shoulder and emergence of a new large mass in S3/S4 (7.5 × 22 mm). Subsequent scans revealed further growth of these tumors in both scapulae and T8. Due to the clinical picture with the absence of pathological lymph nodes, morphology, and the absence of the p63 tumor marker, the giant cell tumor diagnosis was excluded.

**Figure 2:**
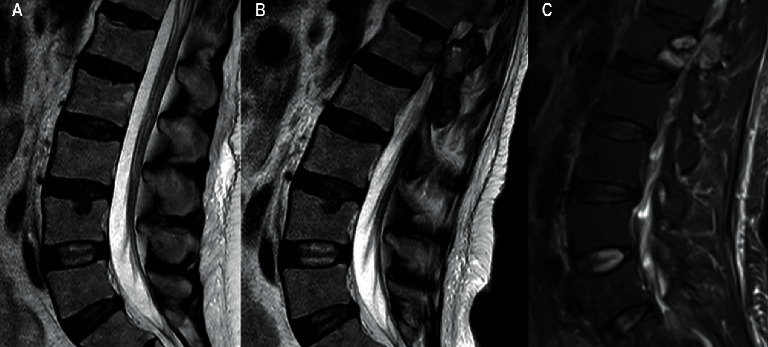
Sagittal spine MRI. (a) T2 weighted sequence from the same investigation as the latest thoracic image showing no lesions in L1. (b) T2 weighted sequence one year later showing tumor in the body and pedicle of L1. (c) T2 weighted Dixon fat suppressed sequence 9 months later showing no further progression of the tumor.

**Figure 3:**
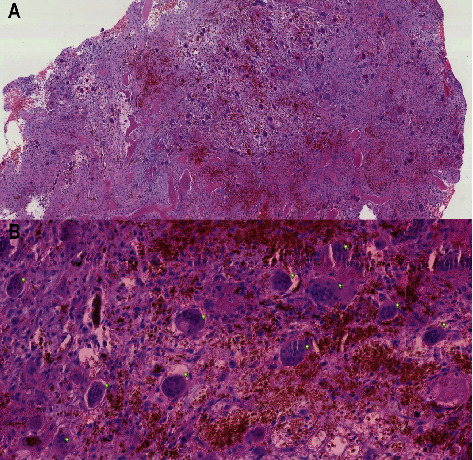
Brown tumor, located at Th12. (a) H&E, x5 histologic features of a giant cell reparative granuloma, in which stromal cells are admixed with osteoclastic giant cells, bone, and areas with hemorrhage. A zonal pattern is seen. Clusters of giant cells aggregate around red blood cells. The giant cells are surrounded by a zone of reactive fibrosis which, in turn, is bounded by the reactive bone. (b) H&E, x100 stromal cells admixed with osteoclast-like giant cells. The multinucleated giant cells, indicated by green dots, aggregate around red blood cells.

Two years later, an MRI revealed a lumbar spine (L1) tumor (Figure 2). A biopsy raised suspicion of a new giant cell tumor. The patient developed severe radicular pain accompanied by weakness of left lower extremity due to neural compression from this lesion. In addition, new spinal lesions developed in corpus of C6 and the right pedicle of L4. Due to the previously described medical history, morphology, negative p63 staining, and the presence of secondary/tertiary hyperparathyroidism (PTH 40–50 pmol/L (1.6–6.0 pmol/L)), a reevaluation of the biopsy material was performed. A giant cell reparative granuloma was described, as shown in Figure 3, and it was concluded that her tumors were indeed BTs. The patient subsequently underwent parathyroidectomy with removal of 3½ parathyroid glands. Postoperative PTH levels were in the normal range 1.0–1.5 pmol/L.

Her pain and symptoms in the lower extremities improved within the first postoperative week. She received short-term dialysis treatment but was discharged on the sixth postoperative day with plasma creatinine approximately 325 micromol/L without further dialysis requirement. There had been neither progression nor regression of the tumor on MRI scanning three months after parathyroidectomy, and her radicular pain and sensory disturbances in the extremities have reduced significantly, with reduced analgetic requirement.

## 3. Discussion

Chronic kidney disease–mineral and bone disorder (CKD-MBD) is a broader clinical syndrome that develops as a systemic disorder of the mineral and bone metabolism due to CKD. It comprises several components including bone pathology, laboratory abnormalities (calcium, phosphorus, FGF23, vitamin D, and PTH), and extra skeletal calcification. Renal osteodystrophy is part of the syndrome of CKD-MBD, being an exclusive term used to describe morphological bone changes associated with CKD. According to the TMV (bone turnover, mineralization, and volume) system of KDIGO guidelines, renal osteodystrophy comprises of OFC, adynamic bone disease, osteomalacia, and mixed uremic osteodystrophy [9, 10]. Brown tumor is a manifestation of OFC in which there is high bone turnover due to increased osteoclast activity caused by hyperparathyroidism. The histologic features include osteoclastic giant cells, which erodes blood vessels and therefore is located around areas of hemorrhage, admixed with the fibrous tissue and woven bone. A characteristic zonal pattern is seen, as the clusters of giant cells aggregate around red blood cells, surrounded by a zone of reactive fibrosis, which, in turn, is bounded by the reactive bone. The location of BT has most frequently been described in the long bones, facial bones, jaws, sternum, ribs, and the pelvic bones [2–4, 6].

As illustrated in our case, misdiagnosis of BT compared to (malignant) neoplastic giant cell tumor of the bone (GCTB) is not uncommon, as both have very similar imaging presentations and histologic findings. These histologic similarities are already described by Jaffe in 1940 [11]. In BT, these multinucleated giant cells are generally arranged in clusters, sometimes with focal hemorrhage and hemosiderin deposition, but cellular atypia and mitotic figures are absent. In comparison, in giant cell tumors, these giant cells tend to be distributed regularly and uniformly with the presence of atypia and mitotic figures. However, there is no accepted histologic criteria presently available to differentiate BT from giant cell tumors [3].

Unfortunately, characterization of the tumors as BTs in our patient was delayed despite morphological changes that did not include neither atypical cells nor regularly distributed multinucleated giant cells in combination with presence of hemorrhage (hemosiderin) and absence of p63 staining. In addition, lack of pathological lymph nodes ruled out the metastatic malignant process. Above all, the clinical presentation (including hyperparathyroidism) in combination with multiple radiologic findings was not consistent with a giant cell tumor of the bone. Our patient had symptoms that may suggest involvement of lumbar spine (L1) with radicular pain in line with a recently reported 42 cases of vertebral BT where pain, paraparesis, and paraplegia were presenting symptoms [3].

Treatment of BTs should be aimed primarily at its prevention by prompt treatment of HPTH with phosphate binders, vitamin D (analogues), calcimimetics, and prolonged dialysis sessions. Although not well studied, Mourad et al. reported improvement of BT lesion of secondary HPTH in a hemodialysis patient treated with high-dose intravenous calcimimetics. While, parathyroidectomy reserved as the rescue option in nonresponsive cases with secondary or tertiary HPTH [6, 9, 12]. In our patient, treatment was delayed by the misdiagnosis; thus, subtotal parathyroidectomy was chosen to be the appropriate treatment modality after review of her case. In review of 127 cases in 2015, Rafael et al. reported on a severe form of OFC and found that medical therapy was only offered to about 10% of cases. Parathyroidectomy was reported to be the most common treatment option, either alone (37%) or in combination with BT resection (22%) [2]. This choice can be debated, although parathyroidectomy is generally recognized as the gold standard for treatment of BTs. This decision was made after consultations with a multidisciplinary team of experts both nationally and internationally. However, our patient refused surgical intervention (resection of the tumor). Reviewing medical options, calcimimetics was ruled out due the longer response time. Denosumab is a neoadjuvant treatment modality in inoperable or locally advanced GCTB, approved by the US food and Drug Administration (FDA) on the basis of several nonrandomized phase 2 trials [13]. While both denosumab and parathyroidectomy have the potential to result in severe hypocalcemia (hungry bone syndrome), denosumab was rejected due to its experimental nature and lack of experience of this treatment option by our local experts. Furthermore, our experts have ruled out GCTB and had the persuasion that our patient had BT.

## 4. Conclusion

Among patients with end-stage renal disease, including dialysis and renal transplant recipients with HPTH and skeletal pain, BT is a relevant differential diagnosis. Early suspicion is essential, and prevention should be the goal of treatment. Early intervention with noninvasive treatment options has good prognosis, as this usually leads to regression of the tumors. Likewise, it prevents the severe consequences such as destructive bone changes of these tumors and compression of adjacent structures.
